# Geographical Variability in the Likelihood of Bloodstream Infections Due to Gram-Negative Bacteria: Correlation with Proximity to the Equator and Health Care Expenditure

**DOI:** 10.1371/journal.pone.0114548

**Published:** 2014-12-18

**Authors:** David Fisman, Eleni Patrozou, Yehuda Carmeli, Eli Perencevich, Ashleigh R. Tuite, Leonard A. Mermel, the Geographical Variability of Bacteremia Study Group

**Affiliations:** 1 Dalla Lana Faculty of Public Health, University of Toronto, Toronto, Ontario, Canada; 2 Hygeia General Hospital, Marousi, Athens, Greece; 3 Division of Epidemiology, Tel Aviv Sourasky Medical Center, Tel Aviv, Israel; 4 Department of Internal Medicine, University of Iowa Carver College of Medicine and Iowa City Veterans Health Care System, Iowa City, Iowa, United States of America; 5 Department of Medicine, Warren Alpert Medical School of Brown University and Division of Infectious Diseases, Rhode Island Hospital, Providence, Rhode Island, United States of America; Centers for Disease Control, Taiwan

## Abstract

**Objective:**

Infections due to Gram-negative bacteria exhibit seasonal trends, with peak infection rates during warmer months. We hypothesized that the likelihood of a bloodstream infection due to Gram-negative bacteria increases with proximity to the equator. We tested this hypothesis and identified geographical, climatic and social factors associated with this variability.

**Design:**

We established a network of 23 international centers in 22 cities. Setting: De-identified results of positive blood cultures from 2007–2011 and data sources for geographic, climatic and socioeconomic factors were assembled for each center.

**Participants:**

Patients at the 23 centers with positive blood cultures.

**Main outcome:**

Due to variability in the availability of total culture volumes across sites, our primary outcome measure was the fraction of positive blood cultures that yielded Gram-negative bacteria; sources of variability in this outcome measure were explored using meta-regression techniques.

**Results:**

The mean fraction of bacteremia associated with Gram-negative bacteria was 48.4% (range 26.4% to 61.8%). Although not all sites displayed significant seasonality, the overall P-value for seasonal oscillation was significant (P<0.001). In univariate meta-regression models, temperature, latitude, latitude squared, longitude, per capita gross domestic product and percent of gross domestic product spent on healthcare were all associated with the fraction of bacteremia due to Gram-negative bacteria. In multivariable models, only percent of gross domestic product spent on healthcare and distance from the equator (ie. latitude squared) were significantly associated with the fraction of bacteremia due to Gram-negative bacteria.

**Conclusions:**

The likelihood of bacteremia due to Gram-negative bacteria varies markedly between cities, in a manner that appears to have both geographic (latitude) and socioeconomic (proportion gross domestic product devoted to health spending) determinants. Thus, the optimal approach to initial management of suspected bacteremia may be geographically specific. The rapid emergence of highly antibiotic-resistant Gram-negative pathogens may have geographically specific impacts.

## Introduction

Gram-negative bacterial infections, including bloodstream infections, are increasingly recognized as exhibiting seasonal trends, suggesting that some variation in the predominance of infecting bacteria may be due in environmental conditions [1]–[4]. In the United States, data derived from adult and pediatric intensive care units demonstrated that *Acinetobacter baumannii* infections were significantly more common in July-October than in November-June, particularly bloodstream infections [3]. Another study demonstrated a dose-response increase in monthly incidence of infection by *Pseudomonas aeruginosa* and *Acinetobacter baumanii* [1]. In a study involving hospitals in four continents, *Klebsiella pneumoniae* bloodstream infections were 1.5 times more common during the warmest months of the year [5].

Such variability may provide clues regarding the potential role of the physical environment in the pathogenesis of bloodstream infections. Environmental contributions to pathogenesis could have important implications in the face of ongoing climate change [6]. Such findings also imply that the optimal approaches to the initial management of bacteremia would vary geographically and temporally, as optimal empirical management of bloodstream infections must balance the risks of inadequate coverage against risks to both individuals and the population associated with excessively broad-spectrum treatment [7], [8]. However, a broad, comparative international assessment of bacteremia risk has not been available to guide such decisions.

We established a network of infectious disease physicians and microbiologists in 23 international medical centers (in 22 cities), and utilized assembled blood culture data to evaluate variability in the occurrence of Gram-negative bacteremia, and to identify geographical and social factors associated with between-region variability.

## Materials and Methods

### Study Design & Setting

An International Steering Committee was established. Hospital-based infectious disease and microbiology experts at different latitudes, and on different continents, were invited to participate in the study. Recruitment of the participating institutions was done by the International Steering Committee. An invitation email was initially sent to 89 centers and a detailed study proposal written by the International Steering Committee was subsequently shared with the contacts that expressed interest in participating in the study. Investigators representing 23 medical centers located in 22 cities (with two hospital groups participating from São Paolo, Brazil) agreed to participate in this study (Table 1). The study was approved by the following research ethics boards: Research Protection Office, Office of Research Administration, Lifespan Hospital System; Hygeia Hospital Scientific Council, Hygeia General Hospital; The Tel Aviv Sourasky Medical Center Institutional Review Board -Helsinki Committee; University of Iowa, Human Subjects Office, IRB-03; Universidad Austral Facultad se Ciencas Biomedicas Comite Institucional De Evaluacion; Research Directorate Southern Health Monash Medical Centre; Joint University of Wollongong and Illawarra Shoalhaven Local Health District Health and Medical Human Research Ethics Committee; Comitê de ética em pasquisa da universidade Federal de São Paulo/Escola Paulista de Medicina; Mount Sinai Hospital Research Ethics Board; Health Research Ethics Board - Health Panel University of Alberta; Dar Al Fouad Hospital Institution Review Board; Univerista Cattolica del Sacro Cuore Comitato Etico; Teine Keijinkai Medical Center Rinri Iinkai; Ethical Committee for Human Research, Faculty of Medicine, Thammasat University; St. John Hospital and Medical Center IRB; and The Washington University in St. Louis Institutional Review Board.

**Table 1:** Location and Characteristics of Participating Centers.

City	Country	Longitude	Latitude	Per Capita GDP ($US)	Income Inequality (Gini Coefficient)	Mean Annual Temperature (°C)	Mean Annual Precipitation (mm)	Percent of GDP on Healthcare	Population Density (individuals/km^2^)
Athens	Greece	23.73	37.98	26,948	0.654	17.4	414	10.2	88
Breda	Netherlands	4.77	51.60	42,194	0.65	9.6	766	9.8	406
Buenos Aires	Argentina	−58.38	−34.60	17,742	0.74	17.7	1215	8.1	15
Cairo	Egypt	31.23	30.06	6,500	0.689	21.8	25	4.7	80
Detroit	USA	−83.05	42.33	48,442	0.801	8.3	849	17.9	67
Diadema	Brazil	−46.61	−23.69	11,719	0.784	19.6	1481	9.0	23
Edmonton	Canada	−113.50	53.55	40,440	0.688	3.9	366	11.3	4
Fortaleza	Brazil	−38.57	−3.77	11,719	0.784	26.7	1642	9.0	23
Goiania	Brazil	−49.25	−16.67	11,719	0.784	23.2	1576	9.0	23
Honda (Hokkaido)	Japan	139.28	36.13	34,294	0.547	8.5	1100	9.5	350
Iowa City	USA	−91.53	41.66	48,442	0.801	10.8	890	17.9	21
Clayton (Melbourne)	Australia	145.12	−37.92	39,438	0.622	15.0	650	8.7	3
Pathumthani	Thailand	100.52	14.02	4,972	0.71	28.1	1418	3.9	135
Porto Alegre	Brazil	−51.23	−30.03	11,719	0.784	20.2	1347	9.0	23
Providence	USA	−71.41	41.82	48,442	0.801	10.0	1198	17.9	388
Rome	Italy	12.50	41.90	32,928	0.609	15.2	804	9.5	206
Salvador	Brazil	−38.47	−12.97	11,719	0.784	25.5	2099	9.0	23
São Paulo	Brazil	−46.62	−23.50	11,719	0.784	18.5	1454	9.0	23
St. Louis	USA	−90.20	38.63	48,442	0.801	14.0	1040	17.9	34
Tel Aviv	Israel	34.80	32.03	31,467	0.677	20.3	532	7.6	352
Toronto	Canada	−79.40	43.65	40,440	0.688	9.2	709	11.3	4
Wollongong	Australia	150.88	−34.43	39,438	0.622	17.6	1329	8.7	3

**NOTE**: Toponymic information is based on the Geographic Names Database, containing official standard names approved by the United States Board on Geographic Names and maintained by the National Geospatial-Intelligence Agency **(** http://earth-info.nga.mil/gns/html/namefiles.htm **)**; per Capita Gross Domestic Product and percent of Gross Domestic Product on Healthcare source is the world bank **(** http://data.worldbank.org/ **)**; climate (temperature, precipitation) source: http://www.weatherbase.com/weather/city; population density source: http://data.worldbank.org/indicator/EN.POP.DNST. Gini coefficients were derived from Davies J.B., Sandstrom S, Shorrocks A, Wolff EN. The level and distribution of global household wealth. NBER working paper #15508; November 2009. Available via the Internet at http://www.nber.org/papers/w15508. Last accessed October 10, 2014.

### Selected datasets

All participating centers provided de-identified results of positive blood cultures from 2007 to 2011 (Table 2). Only the first bloodstream infection per pathogen was included in the analysis. Hence, if a patient had multiple positive blood cultures growing the same pathogen, this would be considered as one bacteremic episode. All pathogens were counted separately if more than one microorganism was isolated from a single blood culture.

**Table 2:** Data Provided by Collaborators for Evaluation of Epidemiology of Bacteremia due to Gram Negative Bacteria.

City	Total Admissions	Positive Cultures	Cultures Growing Gram-Negative Bacteria	Total Cultures	Start Year	End Year	Culture Months	Notes
Athens	49910	1080	474	4863	2007	2011	36	Missing 2008, 2010
Breda	228929	3152	1438	48053	2007	2011	60	
Buenos Aires	35123	895	442	15129	2008	2011	48	
Cairo	44730	914	520	.	2007	2011	60	
Detroit	181143	5168	2415	104500	2007	2011	60	
Diadema	27976	163	103	.	2007	2009	29	June 2007–August 2009
Edmonton	131759	1143	390	.	2007	2011	60	
Fortaleza	11596	180	110	.	2007	2009	21	November 2007–July 2009
Goiania	18204	71	34	.	2008	2009	17	February 2008–October 2009
Honda	58382	1592	837	25380	2008	2011	44	May 2008–December 2011
Iowa City	17037	1418	374	17965	2007	2011	60	
Clayton	838197	5276	2628	154470	2007	2011	60	
Pathumthani	69860	1543	953	69156	2007	2011	60	
Porto Alegre	183679	773	498	.	2007	2009	24	October 2007 to November 2009, combined cultures from two hospitals (Hospital Conceicao and Hospital Santa Casa Porto Alegre).
Providence	177132	4645	1803	.	2007	2011	60	
Rome	290878	5255	2145	78394	2007	2011	60	
Salvador	14863	107	47	.	2007	2009	15	July 2008–January 2010, with gaps.
São Paulo	232268	3942	2263	.	2007	2011	60	Represents combined data from two hospital surveillance systems (the already linked systems of Hospital do Rim e Hipertensao and Hospital Israelita Albert Einstein, and the previously unlinked Hospital Sao Paulo).
St. Louis	256857	9019	2596	64589	2007	2011	60	
Tel Aviv	538708	12609	6312	258959	2007	2011	60	
Toronto	128587	2747	1330	59686	2007	2011	60	
Wollongong	225125	3047	1763	62720	2007	2011	60	

The following microorganisms were considered as contaminants and excluded from the data collection process: *Bacillus sp*, *Corynebacterium sp* (except *Corynebacterium jeikeium*), *Lactobacillus sp*, and *Propionibacterium sp*. Coagulase-negative staphylococci and viridans-group streptococci were included in the analysis if they were recovered from two separate blood cultures, regardless of the time interval between the two positive cultures.

For each participating study center, data was obtained regarding latitude, longitude, mean annual precipitation, mean daily temperature, population density, per-capita gross domestic product and the percentage of gross domestic product in that country that was allocated for healthcare. Data sources for geographic, climatic, and socio-economic covariates are presented in Table 1.

### Statistical methods

Several possible measures of local propensity towards bacteremia due to Gram-negative bacteria are possible; however, the geographic and socioeconomic heterogeneity among participating sites led us to suspect that apparent incidence of bacteremia due to Gram-negative bacteria might be affected by different acuity thresholds for admission to hospital (if patient-days were used as a denominator) and propensity to culture (if total blood cultures were used as a denominator). Thus, we decided to estimate the incidence of bloodstream infection due to Gram-negative bacteria in two ways: first, as all hospitals provided data on the fraction of blood cultures that yielded Gram-negative bacteria and non-Gram-negative bacterial pathogens, we evaluated the Gram-negative bacteremia fraction, defined as Gram-negative bacteremia divided by all bacteremias. Second, as total culture volumes (positive and negative) were available only for a subset of thirteen hospitals, we performed exploratory analyses on the incidence of Gram-negative bacteremia using monthly admissions (available from all participating sites) as model denominators (“offsets”), and adjusting for total culture volumes as model covariates.

For the fraction of bloodstream infections due to Gram-negative bacteria estimates, we converted monthly proportions of positive blood cultures found to contain Gram-negative bacilli to their logits (natural logs of odds), with odds estimated as 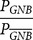, where proportion of bacteremias caused by Gram-negative bacilli is denoted as 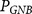 and 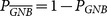. Variance of the logit is approximated as 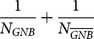, where *N* is the number of isolates [9].

As propensity to culture appeared to be a strong predictor of crude Gram-negative bacteremia incidence, we estimated culture-adjusted incidence of Gram-negative bacteremia per 100 admissions using Poisson regression analysis for the 13 centers for which monthly culture volumes were available [10]. Culture-adjusted baseline incidence density of Gram-negative bacteremia was estimated as the exponentiated intercepts from models that included total culture volume (negative or positive) as a covariate. This quantity can be interpreted as the baseline incidence of bloodstream infections due to Gram-negative bacteria as culture volumes approach zero. We also utilized these models to evaluate culture-adjusted seasonality and waveforms for Gram-negative bacteremia, by adding Fourier transforms to our models such that the estimated incidence of Gram-negative bacteremia 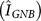 was:
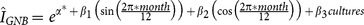
where 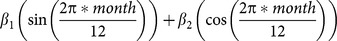


is a Fourier transform representing annual seasonal oscillation, and 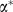 is a composite intercept term that includes both the expected baseline Gram-negative bactereremia culture count, and the monthly admissions offset (denominator) [10], [11]. The 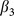 adjusts for total culture submissions. Nonlinear variance estimates were obtained via the delta method. Coefficients from Fourier transforms were utilized to estimate phase as 
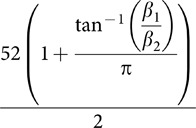
 and amplitude as 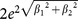 [12]. We approximated standard error for phase and amplitude terms as the sum of standard errors of Fourier terms from Poisson regression models, minus covariance [10], [11].

A significant between-site heterogeneity was identified in the fraction and incidence of bacteremia due to Gram-negative bacteria, we used meta-regression methods [10] to identify geographical, ecological and economic characteristics of cities (latitude, longitude, mean annual temperature and precipitation, population density, national per capita gross domestic product and per capita healthcare spending as a fraction of gross domestic product) that we postulated a priori might explain between city differences in Gram-negative bacteremia. We also evaluated income inequality based on Gini coefficient, a widely used quantitative metric of income distribution [13]. As we postulated that distance from the equator, north or south, might explain changing Gram-negative bacteremia risk, we modeled latitude as a quadratic term [14]. We evaluated the extent to which between-site heterogeneity in waves was explained by model covariates using the tau-squared statistic [15], [16].

## Results

**Figure 1:**
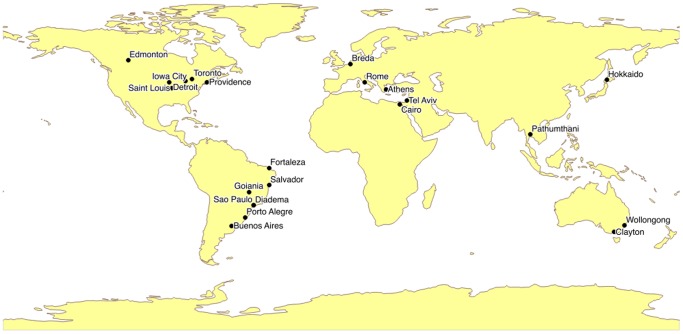
Locations of participating sites.

The locations of 23 participating centers are displayed in Fig. 1 (with two centers located in Sao Paolo, Brazil), and their clinical, geographic, climatic, and socioeconomic characteristics are displayed in Table 1. Cities were arrayed across a range of latitudes spanning 91 degrees (with each degree of latitude equal to approximately 111 km), and a range of longitudes spanning 264 degrees (with each degree signifying a variable distance, depending on latitude, but equal to 85 km at 40 degrees north or south latitude). A total of 1074 months of blood culture data were collected from a total of 23 participating centers in Table 2. Data for two centers located in Sao Paolo were aggregated, such that analyses were based on 22 cities.

**Figure 2:**
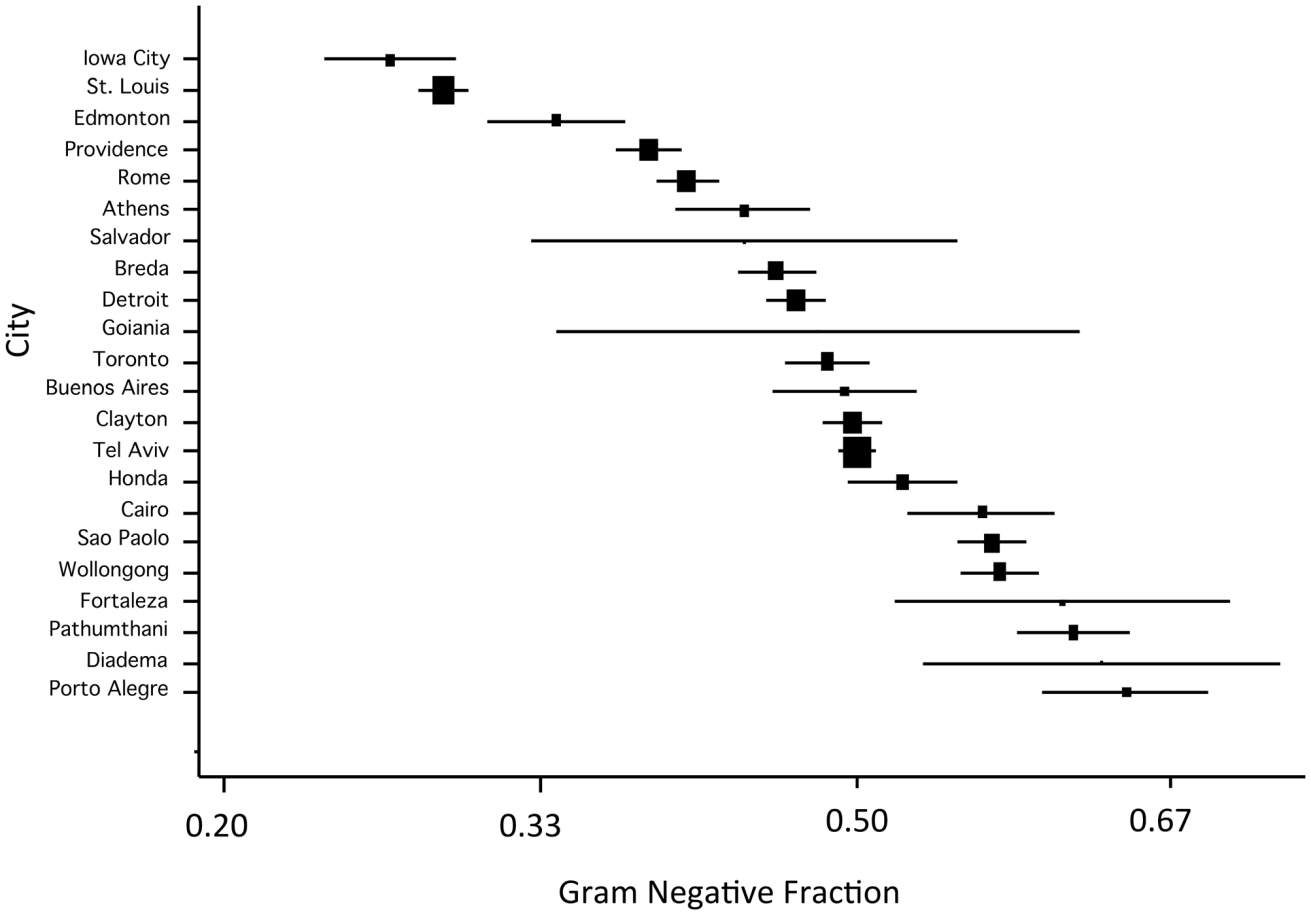
Fraction of bloodstream infections due to Gram-negative bacteria by site (lowest to highest). Note that X-axis is presented on a natural log scale. The area of rectangles is inverse to the variance of log(odds) of bloodstream infection due to Gram-negative bacteria; horizontal lines represent 95% confidence intervals.

The mean fraction of bacteremia associated with Gram-negative pathogens was 48.4% with a range from 26.4% (Iowa City) to 64.4% (Porto Alegre) (Fig. 2). When we assessed the fraction of bloodstream infections due to Gram-negative bacteria via evaluation of logits, we identified extreme heterogeneity (Q-statistic 3547.8 on 21 d.f., P<0.0001).

Culture volumes were highly variable and closely correlated with crude incidence of Gram-negative bacteremia per 100 hospital admissions as well as crude incidence of Gram-negative bacteremia (Spearman's, P = 0.002). Logits for the fraction of bloodstream infections due to Gram-negative bacteria were most closely correlated with intercepts from culture-adjusted models than with crude incidence of Gram-negative bacteria estimates (Table 3). Thus we used fraction of bloodstream infections due to Gram-negative bacteria (primarily, due to completeness of data) and culture-adjusted incidence (secondarily, due to lack of total culture volumes for nine of 23 sites) as our indices of Gram-negative bacterial bloodstream infection risk.

**Table 3:** Spearman Correlation Coefficients and P-Values Comparing Culture Volumes and Metrics for Estimation of Gram NB Risk.

Spearman's Correlation Coefficients (P-value)	Culture Volumes*	Crude Incidence*	Intercept, Culture-Adjusted Model	Logit Fraction of Bloodstream infections Due to Gram-Negative Bacteria
Culture Volumes*	1.0	–	–	–
Crude Incidence*	0.87 (0.0002)	1.0	–	–
Intercept, Culture-Adjusted Model	0.49 (0.11)	0.44 (0.13)	1.0	–
Logit Fraction of Bloodstream Infections due to Gram-Negative Bacteria	0.12 (0.71)	0.16 (0.53)	37 (0.21)	1.0

NOTE: Culture volumes, crude incidence, and culture-adjusted incidence normalized for admission volume. Correlation coefficients for crude incidence and logit GNBF calculated using all 18 sites, whereas coefficients for culture-related metrics restricted to 13 sites for which culture volume was available.

*per 100 admissions.

**Figure 3:**
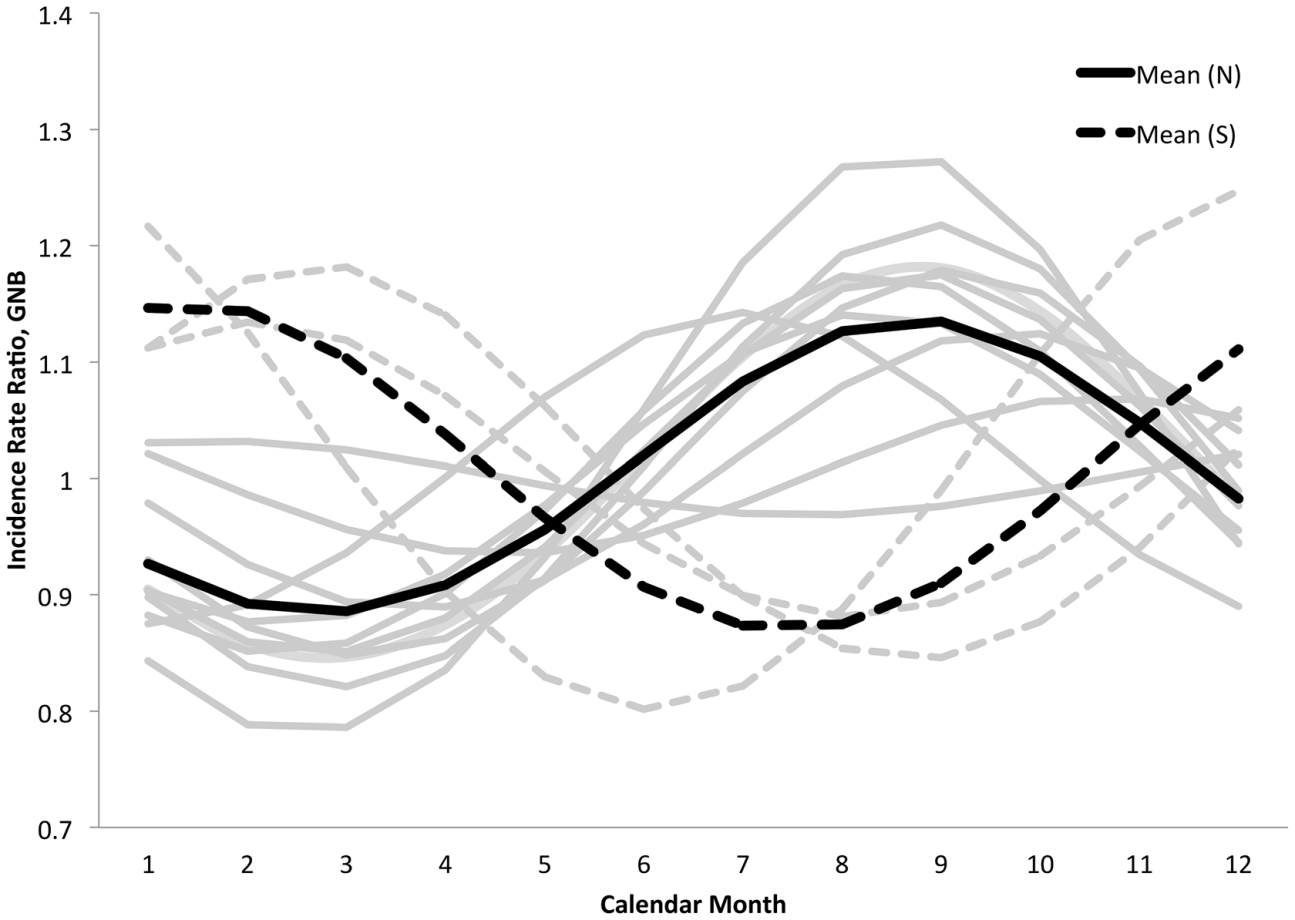
Fitted waveforms for bloodstream infections due to Gram-negative bacteria from Poisson models adjusted for culture frequency for data available at 13 sites. Arithmetic means for northern hemisphere (solid curve) and southern hemisphere (dashed curve) are represented by black curves. Curves for individual sites from northern hemisphere (solid curves) and southern hemisphere (dashed curves) are presented in gray.

**Figure 4:**
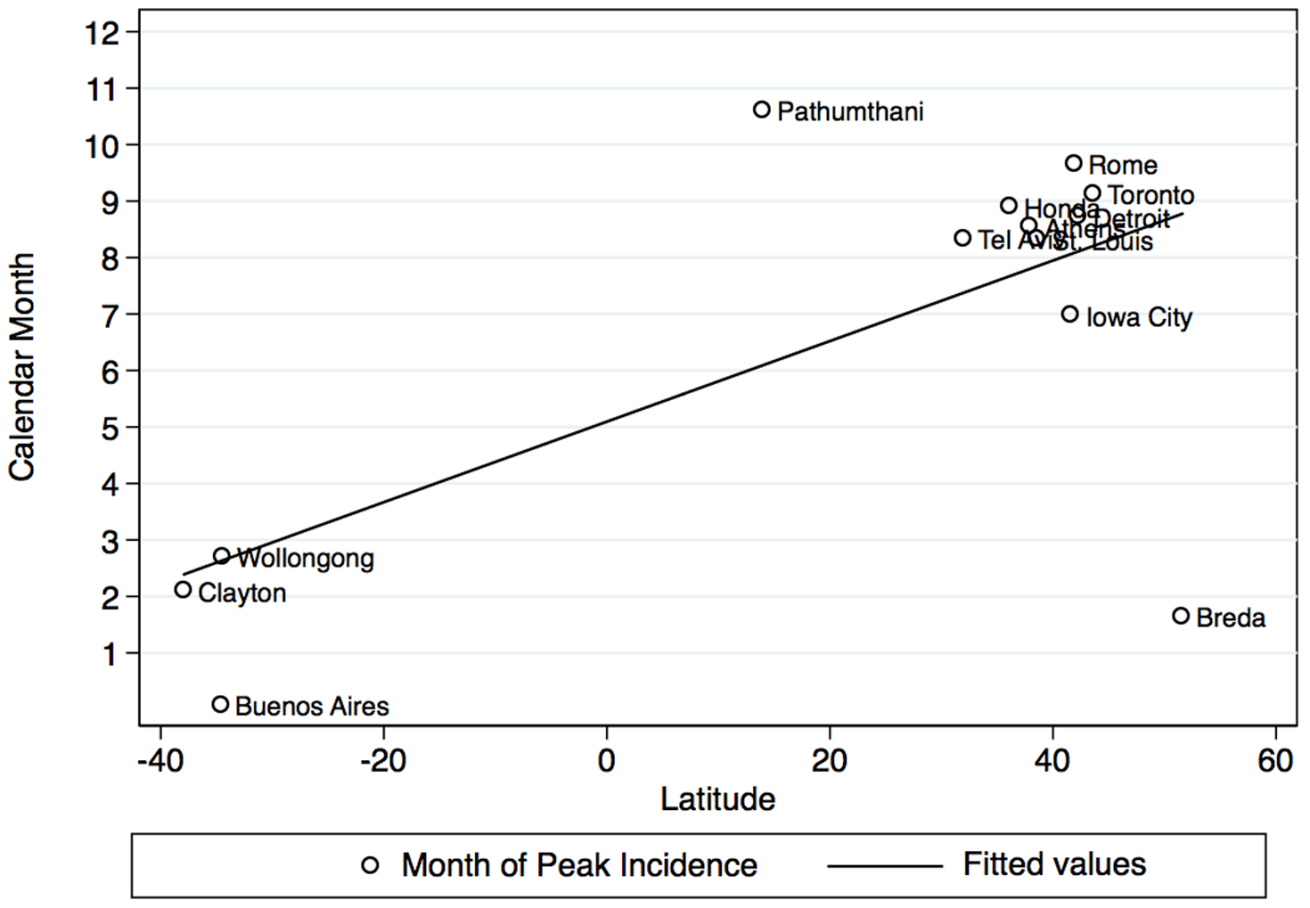
Relationship between peak month of occurrence of bloodstream infection due to Gram-negative bacteria (Y-axis) and latitude (X-axis) (circles). It can be seen that while there is a strong linear overall relationship between month of peak occurrence and latitude, this largely reflects the inversion of summer and winter months in the northern and southern hemispheres (fitted solid black line). Within the northern hemisphere (fitted dashed line) and the southern hemisphere (fitted gray line) there is no relationship between distance from the equator and month of peak incidence.

We evaluated the seasonality of culture-adjusted incidence of Gram-negative bacteremia using Fourier transforms from culture-adjusted incidence models (Fig. 3); although not all sites displayed significant seasonality, the overall P-value for seasonal oscillation was significant (P<0.001). However, the amplitudes of seasonal waveforms were homogeneous (Q-statistic 4.9 on 12 d.f., P = 0.96). Phase terms were heterogeneous (Q-statistic 5675.4 on 12 on 12 d.f., P<0.001). Phase terms remained heterogeneous even when stratified by hemisphere (northern hemisphere (P for heterogeneity <0.001 for phases in both hemispheres). There was a linear relationship between phase (peak month of occurrence) and latitude (P = 0.013) but this relationship simply reflects the inversion of summer and winter months in northern and summer hemispheres, and no relationship was seen between phase and latitude in analyses restricted to the northern or southern hemispheres (Fig. 4).

**Figure 5:**
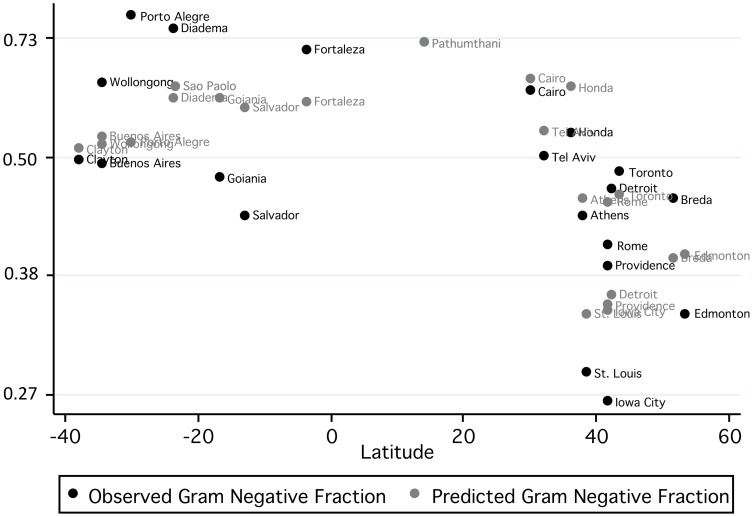
Mean monthly fraction of bloodstream infections due to Gram-negative bacteria (Y-axis) plotted against latitude by study site (black circles). Predicted mean monthly fractions of bloodstream infections due to Gram-negative bacteria, based on a meta-regression model that incorporated latitude-squared, percent of GDP spent on healthcare, and mean annual temperature, are plotted as gray circles. It can be seen that the 3-coefficient model resulted in excellent prediction of Gram-negative bacterial bloodstream infection fraction, and in some cases predictions are sufficiently precise that labels are superimposed.

**Figure 6:**
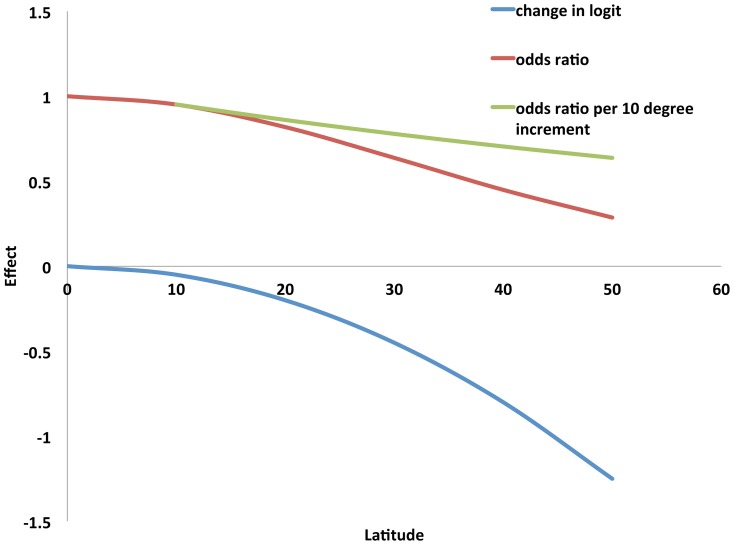
Non-linear effects of latitude on risk of bloodstream infection due to Gram-negative bacteria, among bacteremic individuals, according to distance in degrees from equator. The figure is based on a coefficient for latitude-squared of -0.0003, as presented in Table 4. The log-odds of bloodstream infections due to Gram-negative bacteria (logit, blue curve) is calculated as −0.0003 x latitude^2^, while the odds ratio for GNB (relative to the odds at the equator) is this quantity exponentiated (red curve). The change in odds ratio per 10 degree increment is non-constant by latitude, and is presented as the green curve.

In univariable meta-regression models, several site characteristics, including temperature, longitude, latitude, latitude-squared, per-capita gross domestic product, and percent of gross domestic product spent on healthcare, were associated with the fraction of bloodstream infections due to Gram-negative bacteria. However, in multivariable models, only percent of gross domestic product spent on healthcare and latitude-squared (ie. distance from the equator) were associated with fraction of bloodstream infections due to Gram-negative bacteria at the P<0.05 level (Table 4). Greater explanatory power was seen when mean annual temperature was retained in the model, though this association was not statistically significant (P = 0.135). The resultant multivariable model explained approximately 64% of between-site variation in fraction of bloodstream infections due to Gram-negative bacteria (Fig. 5). No factors explained between-site variation in culture-adjusted intercepts from incidence of Gram-negative bacteremia models (Table 5). It should be noted that the interpretation of latitude-squared coefficients is complex; in our best-fit model for the fraction of bloodstream infection due to Gram-negative bacteria, there is not a constant odds ratio associated with a given change in latitude. Rather, the reduction in Gram-negative bacteremia risk associated with moving from 0 to 10 degrees latitude would be less than that seen moving from 10 to 20 degrees latitude. This relation is presented graphically in Fig. 6.

**Table 4:** Univariable and Multivariable Meta-Regression Models Predicting Log (Odds) of Bloodstream Infection due to Gram Negative Bacteria.

Characteristic	Univariable Models			Multivariable Models		
	Coefficient	95% CI	P-Value	Coefficient	95% CI	P-Value
Longitude	0.003	0.0001 to 0.005	0.042	–	–	–
Latitude	−0.008	−0.013 to 0.003	0.004	–	–	–
Latitude^2^	−0.0004	−0.0006 to −0.0001	0.017	−0.0005	−0.0009 to −0.0007	0.024
Log_10_(Per-Capita GDP)	−0.416	−0.628 to −0.204	0.001	–	–	–
% GDP on Healthcare	−0.079	−0.114 to −0.044	<0.001	−0.077	−0.118 to −0.035	0.002
Income Inequality (Gini Coefficient)	−0.654	−3.290 to 1.981	0.61	_	_	_
Population Density	−0.0003	−0.002 to 0.001	0.72	–	–	–
Mean Annual Temperature	0.040	0.014 to 0.065	0.004	−0.039	−.093 to 0.013	0.148
Mean Annual Precipitation	0.0003	−0.0001 to 0.0007	0.17	–	–	–

**Table 5:** Univariable Models of Factors Influencing Culture-Adjusted Incidence of Bloodstream Infection Due to Gram-Negative Bacteria.

Characteristic	Coefficient	95% CI	P-Value
Longitude	−0.0004	−0.005 to 0.004	0.85
Latitude	−0.0031	−0.017 to 0.010	0.58
Latitude^2^	−0.0003	−0.001 to 0.0004	0.35
Log_10_(Per-Capita Gross Domestic Product	−0.643	−2.267 to 0.981	0.40
% Gross Domestic Product Spent on Healthcare	−0.013	−0.117 to 0.091	0.78
Income Inequality (Gini Coefficient)	2.335	−2.989 to 7.659	0.36
Population Density	−.00008	−0.003 to 0.003	0.95
Mean Annual Temperature	0.013	−.065 to.091	0.71
Mean Annual Precipitation	0.0004	−0.001 to 0.002	0.54

## Discussion

Understanding the epidemiology of bloodstream infection due to Gram-negative bacteria is of great importance as the global spread of highly antimicrobial resistant strains is becoming more prevalent [17]. Previous single state or single country studies have demonstrated a greater incidence of bloodstream infection due to Gram-negative bacteria during warmer months [1], [2], [4], [18], [19], [20], [21]. This observation may reflect optimal growth conditions for many Gram-negative bacteria at 32–36°C. Based on this data, one would suspect that the likelihood of a bloodstream infection due to Gram-negative bacteria compared to Gram-positive bacteria would generally correlate with distance from the equator. Thus, we hypothesized that bacteremia due to Gram-negative bacteria would be most common in medical centers closest to the equator. We assessed this possibility among medical centers at different latitudes in Europe, South America, North America, Australia, and Asia. We confirmed seasonal variability of Gram-negative bacteremia as demonstrated by other investigators. More strikingly, using multivariable, meta-regressive modeling, we demonstrated that the likelihood of bloodstream infection due to Gram-negative bacteria inversely correlated with the percentage of gross domestic product spent on healthcare and distance from the equator measured as latitude-squared. Thus, for bacteremic patients, the likelihood that the infection is due to Gram-negative bacteria is significantly greater in locations closest to the equator, during warmer months of the year, and in locales with a lower amount of gross domestic product spent on healthcare. Lastly, our findings are supported by a recent study that detected a direct correlation between the proportion of the human microbiome made up of Firmucutes (i.e., Gram-positive bacteria) and latitude [22].

What distinguishes our study from previous studies? We included 23 medical centers from different continents across five calendar years, we used a common approach in defining bacteremia, we used logits as a common metric and we controlled for a number of confounding variables in our models. Our data suggests that multiple mechanisms may be at play regarding the likelihood that a bloodstream infection is due to Gram-negative bacteria versus other pathogens, some environmentally-based and others that have economic underpinnings related to healthcare spending. It is hoped that future studies will allow a better understanding of how variables interact leading to the findings we observed. Additionally, one might interpret our seasonal waveform analysis to suggest that bloodstream infection due to Gram-negative bacteria could be less common further from the equator if such infections only predominate during seasonal surges. However, in aggregate, we found oscillatory patterns in Gram-negative bacteremia at all latitudes. We did, on average, observe a flip in waveforms but very heterogeneous and no clear patterns in amplitude that would explain bloodstream infections due to Gram-negative bacteria near the equator via “all year round persistence”. Thus, additional factors such as the physical environment, weather, or topography may have an as yet unexplained impact on this phenomenon.

Our study has limitations. The effects we evaluated were ecological in nature and consequently may be subject to bias. We did not differentiate community-acquired from hospital-acquired bloodstream infection or whether or not they were primary from secondary bloodstream infections. We did not control for in-hospital temperature and humidity and we did not collect data regarding whether or not participating centers had air conditioning for their inpatient wards which could have impacted hospital-acquired bloodstream infections. We also did not control for altitude of the hospitals that participated in this study. The fact that the data collection process did not provide information regarding the frequency with which potential contaminant organisms such as *Bacillus sp.* or a *Propionibacterium sp.* were recovered and excluded or the frequency with which coagulase-negative staphylococci and viridans-group streptococci were excluded from the data collection, in the case that they were isolated only in a single blood culture. It is possible that variations in the blood culture collection practices over time and between centers may have biased our study results by underestimating the true frequency of Gram-positive bacteremias. However, we did address this limitation in data collection by using monthly admissions that were available from all participating sites as a denominator. Also, some Gram-positive bacterial infections, such as those due to *Staphylococcus aureus*, follow a seasonal pattern and they may vary geographically according to distance from the equator; however, we did not assess for that possibility in this study.

Although 23 centers is the largest cohort to date to study this issue, it is not a random sample of geographically distributed hospitals and it still represents a small proportion of all hospitals globally. Additionally, use of blood culture data limits the interpretability of the findings since risk factors for bloodstream infection could represent increased risk of acquisition of the Gram-negative bacteria, increased risk of infection once already colonized, or a combination of both. Lastly, we did not determine which of the many species or groups of Gram-negative bacteria were responsible for the changes noted in this study.

In summary, the likelihood that a bloodstream infection is due to Gram-negative bacteria compared to other bacteria correlates with distance from the equator and healthcare spending. We hope that future studies will build on these observations adding to the existing knowledge regarding the epidemiology of Gram- negative bacteremia and assist in the control of such infections in different parts of the world.
