# Correction: Geographical Variability in the Likelihood of Bloodstream Infections Due to Gram-Negative Bacteria: Correlation with Proximity to the Equator and Health Care Expenditure

**DOI:** 10.1371/journal.pone.0122435

**Published:** 2015-03-23

**Authors:** The PLOS ONE Staff

The individuals who comprise the Geographical Variability of Bacteremia Study Group should be included in the author byline instead of the Acknowledgements. The authors and their affiliations are listed below.

Rodolfo E. Quirós, Division of Infectious Diseases, Prevention and Infection Control Service, Hospital Universitario Austral, Buenos Aires, Argentina

Viviana Vilches, Laboratory of Microbiology, Hospital Universitario Austral, Buenos Aires, Argentina

Tony M. Korman, Monash Infectious Diseases, Monash Health, Clayton, Australia

Spyros Miyakis, The Wollongong Hospital, Wollongong, Australia

Craig S. Boutlis, The Wollongong Hospital, Wollongong, Australia

Alistair B. Reid, The Wollongong Hospital, Wollongong, Australia

Ana Cristina Gales, Laboratório Especial de Microbiologia Clínica, Division of Infectious Diseases, Universidade Federal de São Paulo, São Paulo, Brazil

Lygia Schandert, Laboratório Especial de Microbiologia Clínica, Division of Infectious Diseases, Universidade Federal de São Paulo, São Paulo, Brazil

Rafael Affini, Laboratório Especial de Microbiologia Clínica, Division of Infectious Diseases, Universidade Federal de São Paulo, São Paulo, Brazil

Antonia Machado Oliveira, Laboratório Especial de Microbiologia Clínica, Division of Infectious Diseases, Universidade Federal de São Paulo, São Paulo, Brazil

Alexandre R. Marra, Hospital Israelita Albert Einstein, São Paulo, Brazil

Luis Fernando Aranha Camargo, Hospital Israelita Albert Einstein, São Paulo, Brazil

Michael B. Edmond, Virginia Commonwealth University Medical Center, VCU Medical Center, Richmond, Virginia

Luci Correa, Hospital Israelita Albert Einstein and Hospital do Rim e Hipertensao, São Paulo, Brazil

Teresa Cristina Teixeira Sukiennik, Hospital Santa Casa de Porto Alegre, Porto Alegre, Rio Grande do Sul, Brazil

Paulo Renato Petersen Behar, Hospital Conceicao, Porto Alegre, Rio Grande do Sul, Brazil

Evelyne Girão, Hospital Walter Cantidio, Fortaleza, Ceara, Brazil

Carla Guerra, Hospital de Diadema, São Paulo, Brazil

Carlos Brites, Hospital Espanhol, Salvador, Bahia, Brazil

Marta Antunes de Souza, Hospital das Clinicas de Goiania, Goiania, Goias, Brazil

Allison J. McGeer, Mt. Sinai Hospital, Toronto, ON, Canada

Stephanie Smith, Division of Infectious Diseases, University of Alberta, Edmonton, Alberta, Canada

Amani A. El Kholy, Clinical Pathology Department, Faculty of Medicine, Cairo University (Kasr Al Ainy), Dar Al Fouad Hospital, Cairo, Egypt

George Plakias, Hygeia General Hospital, Marousi, Athens, Greece

Evelina Tacconelli, Division Infectious Diseases, Internal Medicine, Tübingen University Hospital, Tübingeneve

Hitoshi Honda, Department of Infection Prevention, Tokyo Metropolitan Tama Medical Center, Tokyo, Japan

Jan Kluytmans, Amphia Hospital Breda, Laboratory for Microbiology and Infection Control, Netherlands

Anucha Apisarnthanarak, Division of Infectious Diseases, Thammasat University Hospital, Pratumthani, Thailand

Mohamad G. Fakih, Infection Prevention and Control Department, St John Hospital and Medical Center, Grosse Pointe Woods, Michigan

Jonas Marschall, Department of Infectious Diseases, Bern University Hospital and University of Bern, Bern, Switzerland

Anthony J. Russo, Barnes Jewish Hospital, St Louis, Missouri
